# Influences of physical stimulations on the migration and differentiation of Schwann cells involved in peripheral nerve repair

**DOI:** 10.1080/19336918.2025.2450311

**Published:** 2025-01-16

**Authors:** Qingyan Sun, Xiaodan Mu, Qi Gao, Juncheng Wang, Min Hu, Huawei Liu

**Affiliations:** a Department of Stomatology, The First Medical Center, Chinese PLA General Hospital, Beijing, China; b Chinese People’s Liberation Army (PLA) Medical School, Beijing, China; c Department of Stomatology of Air Force Hospital in the Southern Theater, Guangzhou, Guangdong Province, China

**Keywords:** Durotaxis, galvanotaxis, magnetotaxis, peripheral nerve repair, Schwann cells

## Abstract

Peripheral nerve injury repair has always been a research concern of scientists. At the tissue level, axonal regeneration has become a research spotlight in peripheral nerve repair. Through transplantation of autologous nerve grafts or other emerging biomaterials functional recovery after facial nerve injury is not ideal in clinical scenarios. Great strides have been made to improve facial nerve repair at the micro-cellular level. Physical stimulation techniques can trigger Schwann cells (SCs) to migrate and differentiate into cells required for peripheral nerve repair. Classified by the sources of physical stimulations, SCs repair peripheral nerves through galvanotaxis, magnetotaxis and durotaxis. This article summarized the activation, directional migration and differentiation of SCs induced by physical stimulations, thus providing new ideas for the research of peripheral nerve repair.

## Introduction

The peripheral nervous system (PNS) is a network of motor and sensory nerves [1]. A peripheral nerve injury (PNI) occurs when the motor or sensory nerves are damaged, leading to the loss of function and clinical symptoms of pain, weakness, numbness and trouble walking [2]. Compared to the central nervous system (CNS), PNS can regenerate after injury. An anatomical integrity of peripheral nerves is necessary for axonal regeneration. Axonal regeneration occurs spontaneously and naturally in short nerve gaps, but microsurgery is essential to axonal regeneration over long nerve gaps [3]. It is proved that after nerve injuries, natural axonal regeneration occurs over a maximum of 4-cm human residual gap [4]. Autograft reconstruction is the gold standard for treating PNI, although a physical alignment can only partially restore nerve functionality. A tissue-level repair after PNI is expected. Parkinson et al. found the formation of nerve bridges in mice after peripheral nerve transection injury, which is attributed to the misdirection of regenerating axons that barely penetrate the distal end of the transected nerve. Eventually, a neuroma and scars form with a bundle of axons, leading to severe pain [5]. A micro-level repair of PNI achieves the goal of functional recovery to the greatest extent. The direct migration of SCs to the injured site is a pivotal link in repairing PNI.

**Figure 1.**
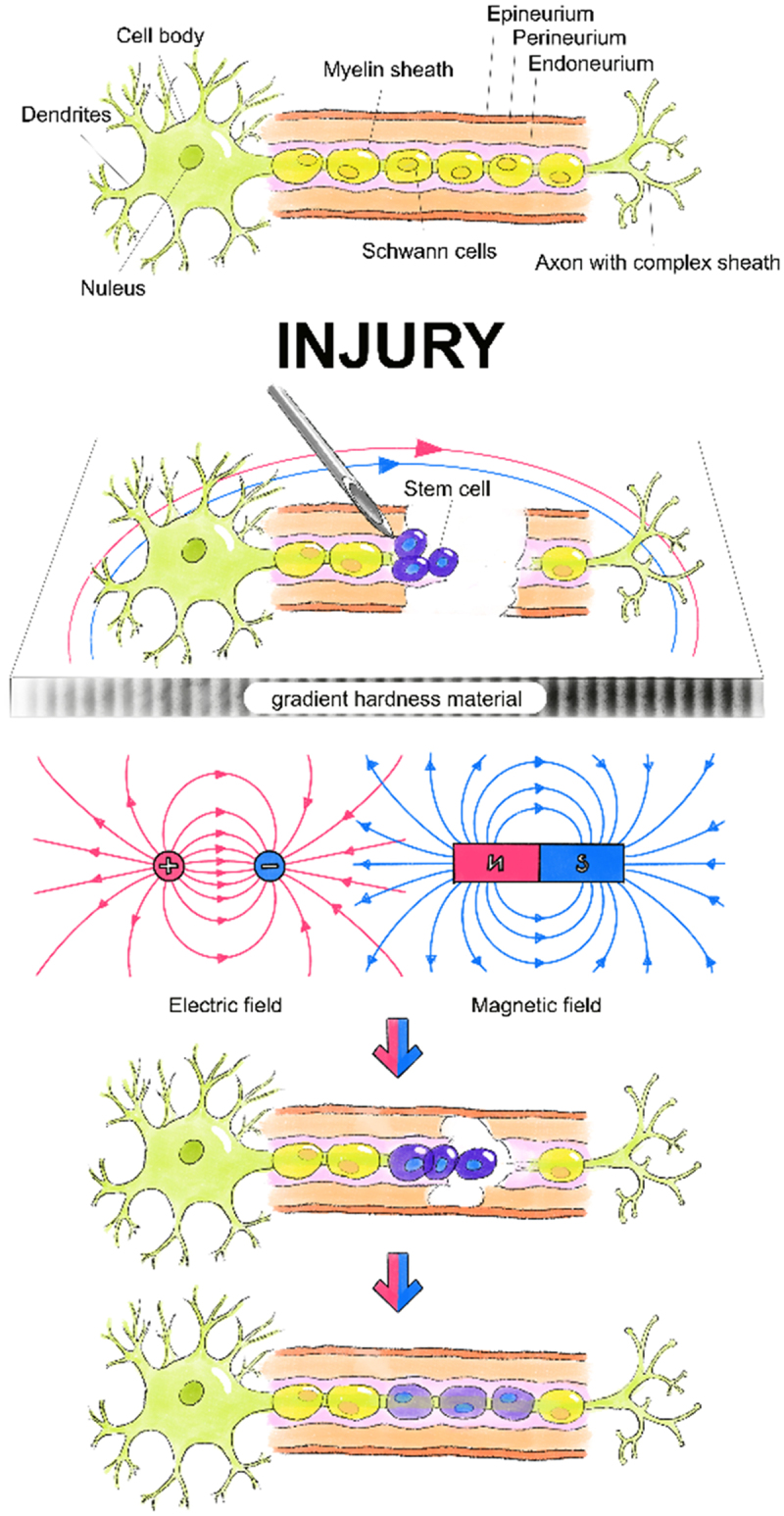
Neural repair is completed by Schwann cells directed migration and differentiation under the influence of physical environments such as electric field, magnetic field and environmental hardness.

In this review, we briefly introduced the cellular mechanism of PNI and SCs-mandated repair. Physical factors influencing the migration and differentiation of SCs involved in PNI repair were thoroughly explored (Figure 1).

## Anatomy of peripheral nerves

Peripheral nerves are composed of endoneurium, perineurium, epineurium, endoneurial fluid, blood vessels, fasciculi, and axons. Endoneurium is a layer of connective tissue surrounding the nerve fiber. The perineurium surrounds the fascicle of nerve fibers, and the epineurium is the outermost layer that wraps the peripheral nerve. Neurons and blood vessels bundle together to form a fasciculi. Axons are the elongation of neurons that transmit information [6].

## Repair processes after PNI

Nerves in the environment and SCs synergistically promote myelination and repair of sciatic axon injury [7]. SCs in the distal stump, which are originally wrapped by surrounding myelin sheath, undergo extensive reprogramming to support axon repair [8]. Wallerian degeneration quickly responds to PNI, leading to the breakdown of both axons and myelin in the distal portion [9]. Besides exerting a direct stimulation on Wallerian degeneration [10], SCs themselves dedifferentiate and transform into longer, bipolar, branched reparative SCs that partially overlap with neighboring cells within their basal lamina tubes. During the injury-induced conversion into reparative SCs, a more motile, proliferative and invasive cellular phenotype also develops. Dedifferentiated reparative SCs generate bands of Büngner by proliferating and migrating which slowly sprout axons by 1-4 mm per day following PNI [11]. Finally, repair phenotype SCs form a one-to-one relationship with caliber axons then myelin sheath wrap SCs, transforming SCs type into normal and completing peripheral nerve repair [12].

## Directional migration of SCs and influencing factors

**Figure 2.**
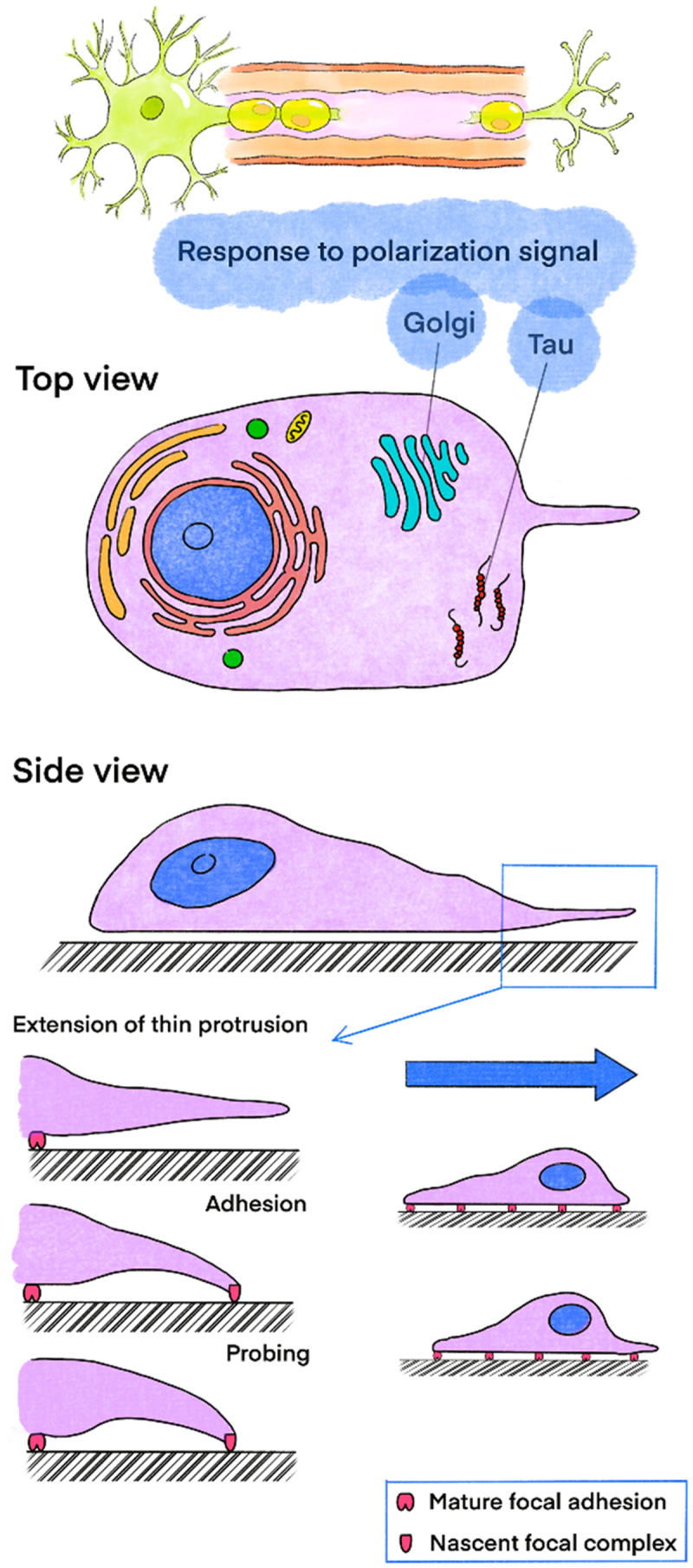
The process of cell migration is manipulated by Golgi, Tau and protrusion.

Cell migration is a process by which external cues bias the intrinsic polarity to orient cells [13]. Polarization is the initial response to cell migration, in which the Golgi apparatus and microtubules are involved. The microtubule-associated protein tau (MAPT) plays an essential role in microtubule assembly and stability, as well as the functional maintenance of the nervous system. In a tau-knockout mouse model, SCs present a higher proliferative rate, but lower capacities of migration and debris clearance after sciatic nerve injury. In response to polarity signals, cells extend protrusions to follow the guidance primarily driven by actin polymerization. The leading edge of lamellipodia forms new adhesive contacts with the transmembrane receptors of the extracellular matrix (ECM) or adjacent to achieve stabilization. Codirectional adhesions serve as traction points during forward cell migration, while adhesions at the rear disassemble. (Figure 2) Adhesion is primarily mediated by integrins, as specific transmembrane receptors are expressed in each cell type [14]. A cell cycle-regulating protein kinase, Cdc2 depends on cyclin to promote cell migration in PNI [15].

Rac1, Cdc42, and RhoA are small Rho GTPases activated by the GTP-bound and GDP-bound cycles in the PNS. Although act as lamellipodia and filopodia-related complexes, they independently induce focal adhesion. Increased activation of Rac1 via beta1 integrin transforms the movement and morphology of SCs to allow axonal sorting and myelination by extending radial membranes [16]. Low-level Rac1 activation favors directional cell migration, while level Rac1 activation facilitates cell aggregation [17]. Cdc42 induces the formation of filopodia by altering the cytoskeleton [16]. RhoA assembles stress fibers and forms focal adhesions [16]. However, small Rho GTPases are activated sequentially by varied stimuli during cell migration and further activate actin to influence cell morphology [16,18,19].

## Galvanotaxis

Electric stimuli pose an evident effect on tissue development and regeneration. A constant electric field at 140 mV/mm is spontaneously formed during nerve repair [20]. Cells are certified to react to an electric field by directional migration, which is known as galvanotaxis [21]. In varied electric fields, experimental biological subjects show specific preferences toward the cathode or anodal [22; 23] [24]. Under exposure to an electric field, cell-cell interactions and the fluctuating direction of cell migration promote directed collective migration [25]. Electric currents induce endothelial cells, bone marrow mesenchymal stem cells [26], and human dermal fibroblasts to migrate directionally. The migration rate of nucleus pulposus-derived stem cells (NSPC) can increase voltage-dependent from 0 to 250 mV/mm, but maintain between 50 mV/mm and 100 mV/mm [27].

## Cellular change affected by electric field

The polarity and movement of cells have a strong relationship with Golgi polarization during galvanotaxis [28; 29] [30; 31]. Persistent electric stimulation induces directional migration by establishing and maintaining cell polarity [32]. Exposed to an electric field, cells undergo reorientation and polarization of centrosome and Golgi apparatus, thus moving toward the same direction [28;29] [23; 31]. Rac1 is an important regulator of plasma membrane potential (Vm) depolarization. Rac1 fine-tunes cell migration in response to ionic and/or electric field changes within the local microenvironment. Through Nav 1.5-dependent Vm depolarization, phosphatidylserine can colocalize to activate Rac1 at the leading edge of migrating cells [33]. Upregulation of α-tubulin acetylation stimulates cell migration in a bio-intensity electric field [34]. Field strength has no significant correlations with migration velocity and voltage-gated calcium channels (Cav3.2 and Cav1.4) within particular limits [35].

Several signaling pathways participate in cell movement in an electric field. Dependent on the activity of actin, guanylyl cyclase (GCase)-related and PI3K-related signaling pathways act on the leading edge of cells to partially decrease the efficiency of galvanotaxis [36]. Cell migration of *Dictyostelium* can be reversed by genetic upregulations on GCases and cyclic guanosine monophosphate (cGMP)-binding protein C (GbpC), combined with the inhibitions on phosphatidylinositol-3-OH kinases (PI3K). Direct current electric field (DCEF) drives the migration of microglia by regulating the ERK/GSK3β/cofilin signaling pathway. Similar to the protein kinase C activator PMA. LiCl is a GSK3β inhibitor that steers the galvanotaxis of microglia. In contrast, PMA reverses cell migration through the ERK/GSK3β/cofilin axis [37]. Exposure to an electric field can promote keratinocyte migration by downregulating CD9 via the AMPK pathway [38]. TMEM87a/Elkin1 modulates cellular adhesions, melanoma cell migration and cell-cell interactions through the PIEZO1-independent mechanoelectrical transduction pathway [39]. An electric field polarizes F-actin to achieve cell migration via the ADAM17/HB-EGF/EGFR signaling pathway. Phosphorylated EGFR also stimulates collective migration, which is further validated by the finding that the ADAM17 inhibitor TAPI-2 significantly decreases the phosphorylation of EGFR and shedding of HB-EGF [40]. Immortalized nontumorigenic human epidermal (HaCaT) cells exposed to electric fields demonstrate a stronger migrative rate, which may involve the activation of the Erk1/2 signaling pathway [41]. Although the expression of TRPM7 is positively correlated with the electric field strength, upregulation of TRPM7 is considered as a compensatory mechanism, due to the bare influence of TRPM7 and channel inhibitors on cell migration. Mg^2+^ block the Ca^2+^-influx and stimulates store-operated Ca^2+^ release to reduce the speed of cell migration [35].

## Neural differentiation is correlated with electric stimulation

Neuronal differentiation can be induced by electric stimuli [42,43]. Graphene-based electrodes trigger mesenchymal stem cells to differentiate into SC-like cells, without the need for additional chemical stimulators [44]. Electric stimuli significantly promote proliferation, neuronal differentiation and neurite elongation of neural stem cells (NSCs) planted on the surface of piezoelectric materials [45]. Through direct current pulse stimulation in mice, neural stem and progenitor cells (NPCs) can be differentiated into neurons, astrocytes and oligodendrocytes [46]. Stem cells stimulated with a nanosecond pulse electric field at 5kV and 10kV can differentiate into SC-like cells within 3–4 days [15]. Human mesenchymal stem cells differentiate into neural progenitor cells within 24 h under the stimulation of an electric field at lower than 1.0 V for 200s and shorter, or into neurons on day 3 under three-electrode electrostimulation [47]. Cranial neural crest cells (CNCCs) exhibit a robust directional migration in response to physiological electric field (<30 mV/mm) toward the anode in a voltage-dependent manner and the rate of migration and displacement increases with field strength [48].

However, improper electric stimuli result in the oncogenesis or death of cells. For example, an electric stimulation at a voltage bias of ±0.5 V and ±1.0 V does not show any adverse effect on cell viability and proliferation but at a voltage of ±1.5 V, it causes an upsurge in cell death [47]. Neuronal differentiation of PC12 cells is greatly accelerated by an electric field at 30–80 mV/mm but inferiorly performed under electric stimulation at other intensities [42,43]. An electric field at a high intensity breaks cell membrane fragments [49]. Exposure to external electric stimulation places SCs at a high risk of transformation into Schwannoma, a benign tumor of the PNS. Neuronal differentiation of PC12 cells is greatly accelerated by an electric field at 30–80 mV/mm but inferiorly performed under electric stimulation at other intensities [42,43]. An electric field at a high intensity breaks cell membrane fragments [49]. Exposure to external electric stimulation places SCs at a high risk of transformation into Schwannoma, a benign tumor of the PNS [50].

Dielectrophoretic behaviors, such as membrane permittivity, conductivity, and cytoplasm conductivity, show changes after cell differentiation induced by electric stimuli [51]. The voltage-gated calcium channel (Cav^2+^) plays an especially important role in the neuronal differentiation of NSCs under electrical stimuli [52]. Ion channel gating of the cytomembrane mediates the concentrations of potassium and sodium, which is primarily responsible for cell differentiation induced by electrical stimuli [53,54]. Ca^2+^ and calmodulin-kinases activate neural differentiation and expedite neurite development under a high electric field [55,56]. A growing number of neurite protrusions, after an electrical stimulation, further promote cell differentiation [57]. An artificially controlled piezoelectric potential is found to stimulate stem cell differentiation, with cell traction as a loop feedback signal [58]. RNA sequencing validated that autophagy is involved in NSC-derived neuronal differentiation under electric stimuli. Excessive ES currents can enhance neuronal autophagy. Either insufficient or excessive autophagy contributes to neurite degeneration. Reactive oxygen species (ROS), generated by an increased intracellular mitochondrial membrane potential (MMP, ΔΨ M), execute an essential role in neuronal differentiation, and excessive ROS production closely links with neurodegeneration and nerve cell injury [47]. Under an electrical stimulation at nanoscale topography, neuronal cells present lower viability, neurite outgrowth and electrophysiological activity, in which autophagy signaling is involved [59]. A series of key molecules are involved in neuronal differentiation induced by electric stimuli. Synaptic transmission, plasticity, and growth are strongly modulated by brain-derived neurotrophic factor (BDNF) [60]. Both the concentration and electrophysiological activity of BDNF have a close relationship with neural differentiation [59]. Glial cell line-derived neurotrophic factor (GDNF) improves neurobehavioral recovery by promoting endogenous tissue sparing, enhancing the electrical integration of transplanted cells, and facilitating synaptic interactions [61,62]. Exposure to electric fields significantly triggers neuronal differentiation by upregulating the mRNA and protein levels of Ascl1. Overexpression of Hes1 promotes the proliferation but inhibits the differentiation of neurons induced by electric fields [63]. Both microtubule-associated protein 2 (MAP2) and β-tubulin III, markers of the end stage of differentiation, are upregulated by electrostimulation, leading to postmitotic neurons [64]. Other differentially expressed genes under an electric stimulation involved in neuronal differentiation include upregulated MAP2, neurofilament heavy chain (NF-H) and Ca^2+^, and downregulated glial fibrillary acidic protein (GFAP) and vimentin [65,66]. The PI3K/AKT pathway is associated with stem cell neural differentiation [67]. Nanosecond pulsed electric fields demethylate the promoters of stem cell pluripotency genes OCT4 and NANOG through an instantaneous downregulation of DNA methylation transferase 1 (DNMT1), allowing stem cells to differentiate [68]. A collective effort with mechanical and electrical stimuli superiorly generates induced pluripotent stem cell (iPSC)-derived neurons by mediating the RhoA/ROCK signaling and de novo production of ciliary neurotrophic factor (CNTF), and these neurons can be based on to study the neurotransmitter release in human neurons [69].

## Magnetotaxis

Magnetotaxis is a process by which cells migrate, proliferate and differentiate in response to magnetic fields [70,71]. Magnetic stimuli intervene in cell-membrane processes via ionic channels, cell receptors, and the membrane potential [72–78]. Sensitized by external magnetic and mechanical forces, and transduced into intracellular biochemical signaling, cells further activate genes associated with cell migration and differentiation [79]. Exposure to a magnetic field can enhance the viability of SCs after transplantation and promote nerve regeneration and functional recovery [80]. Consistently, magnetically driven signals promote nerve regeneration by maintaining SCs in a reparative phenotype [81]. SCs are highly associated with the elongation of neurites. Magnetic stimulations regulate the growth of axons, presumably due to growth cones at the neurite tips in response to guidance cues [82].

## Different intensities of magnetic stimuli result in various cell activities

Magnetic treatments can regulate cell behaviors in various ways. SCs orient parallel to the magnetic fields after 60 h of 8-tesla magnetic stimulation [83]. When mixed with collagen, in contrast, they move perpendicular after a 2-h exposure to the magnetic field [84]. Magnetic stimulation at 3e50 mT results in positive effects on cell activity and proliferation, but opposite trends are observed after exposure to a magnetic field at 4e15 mT [26,85]). In cells exposed to parallel and perpendicular magnetic fields at 70 mT, the formation of lamellipodia and filopodia accelerates to promote cellular migration [86]. Cell proliferation can be stimulated by high magnetic fields (70 mT), but inhibited by low parallel magnetic fields (30 mT). It is currently believed that high magnetic fields can change the membrane potential by regulating the movement of paramagnetic or diamagnetic species [87]. However, how high magnetic fields alter cell behaviors has not been well defined.

At both atomic and molecular levels, magnetotaxis mediates tissue regeneration through formatting and rearranging microtubules and actin filaments [86]. SCs exposed to a magnetic field have a stronger ability to directly migrate along the magnetized force with the assistance of the activated integrin [88,89]. Magnetic stimulations at a moderate strength can inhibit cancer cell migration and stemness by upregulating genes associated with oxidative stress, including hyaluronan receptor (CD44), SRY-box transcription factor 2 (Sox2), and cell myc proto-oncogene protein (C-myc) [90]. Moreover, secretions of protein BDNF, GDNF, neurotrophin-3 (NT-3), and VEGF are also boosted by magnetic fields, thus promoting cell adhesion, spreading, and proliferation [91]. Inconsistent findings, however, show that the expression level of NT-3 does not change after exposure to a pulsed magnetic field [92]. Liu et al. indicated that repeated magnetic stimulations of the proliferation of NPCs by downregulating p21 and upregulating miR-106b. The proliferation and migration of human dental pulp stem cells (DPSCs) can be facilitated through upregulating phosphorylated c-Jun N-terminal kinase (p-JNK), P38 and extracellular signal-regulated kinase (ERK) with magnetic fields [93]. In addition, upregulating FGF-2, TGF-β and VEGF in a static magnetic field at 1 mT can increase the proliferative rate of DPSCs. MMP-1 and MMP-2 also participate in the migration of DPSCs enhanced by a static magnetic field at 1 mT [94]. The expression level of VEGF barely changes in cells induced with magnetic fields at a frequency of 15 hz versus an intensity of 1.8 mT [95].

Cell differentiation is influenced by the type, duration, intensity and frequency of magnetic fields. In the absence of other neural-inducing factors, the effect of electromagnetic induction on neuronal differentiation of bone marrow mesenchymal stem cells (BMSCs) and adipose-derived mesenchymal stem cells (ADMSCs) involves the upregulation of certain genes/proteins and the increase of Ca^2+^ intracellular flow [96,97]. A short-term (2 days) intermittent exposure to a low magnetic field increases adipogenesis, while a long-term (7 days) intermittent exposure combined with continuous exposure favors osteogenesis. In addition, osteogenesis can also be stimulated by short-term intermittent exposure to a high magnetic field at 21.6 mT [98]. Low frequency and intermittent theta-burst stimulation (iTBS) can promote the generation of mature neurons from human iPSCs, while iTBS alone may promote synapse formation during differentiation. Unlike low-frequency, high-frequency stimulation promotes the differentiation into glutamatergic neurons [99]. Various signaling pathways are responsible for neuronal differentiation during magnetotaxis. Intracellular RAS-dependent signals are activated to facilitate the cascades of neuronal differentiation [100]. The non-canonical TGF-β-Akt and TGF-β-Erk1/2 pathways, but not the canonical SMAD pathway, are activated to enhance the differentiation of oligodendrocyte progenitor cells in a low magnetic field [101]. β-catenin immediately suppresses the self-renewal of mouse cortical neural precursor cells (NPCs) but promotes neuronal differentiation by activating the Wnt signaling pathway [102].

## The various effects of magnetic nanoparticles on neuron

The attachment of magnetic microbeads to neuronal membranes can generate extracorporeal mechanical forces to achieve a stretch-growth of axons [103]. Fe_3_O_4_ magnetic nanoparticles (MNPs) have been designed as microbeads [104]. Cells are normally magnetized after absorbing magnetic nanoparticles (MNPs). Magnetic field combined with MNPs can elongate cells and nuclei, and then induce differentiation [105]. Signaling transduction for magnetotaxis is consistent with that for cellular stress response. The tensile force that magnetaxis exerts on the axons can direct the growth of neurons. Iron oxide concentration increases from 0% to 10% to accelerate the process neural differentiation process [106]. Quite different from sole exposure to magnetic field, magnetic field combined with specifically designed Fe_3_O_4_ polymeride significantly drives cell adhesion and migration by upregulating FGD2 and PODXL, as well as activating the MAPK cascade [104]. Through targeting and upregulating TREK1, MNPs participate in neuronal cell stress response by activating the c-Myc/NF-κB signaling pathway. Similarly, in cells induced with mechanical stimulation, TREK1 functions as a regulator in the NF-κB signaling pathway to promote axon production. However, no evidence supports the regulatory effect of TREK1 on neuronal differentiation [107].

Cell viability can be suppressed by a high concentration of magnetic iron oxide particles, especially in neurons sensitive to the environment [108]. It is found that cell viability and activity are gradually suppressed after exposure to anionic MNPs at a concentration increasing from 0.15 mm to 15 mm of iron [108]. Some iron oxide nanoparticles have been certified as clinically-used contrast agents [109,110], but most of them require clinical validation [111,112].

## Durotaxis

Durotaxis is a process in which cells migrate in response to gradients in the stiffness of their extracellular matrix (ECM). Regardless of the stiffness of the environment where cells are initially domesticated, all cells migrate to more rigid substrates by changing the mechanical stiffness input. Durotaxis is first observed in single cells, as shown by durotactic behaviors in extracorporeal cells like fibroblasts, stem cells, smooth muscle cells, isolated human mammary epithelial cells (MCF-10A), and chondrocytes [113]. However, *in vivo*, durotaxis remains controversial [114]. Some believe that a directional cellular motion is related to gradients of dynamic stiffness, and chemical and mechanical factors *in vivo* [115]; while others suggest that chemotaxis is a leading cause of directed migration *in vivo* following chemical gradients [115–118]. Considering the inconsistency between *in vitro* and *in vivo* durotaxis, great efforts have been made to identify the signals responsible for cell migration in response to substrate rigidity and mechanical forces. Integrin-based focal adhesions have been established as a regulator of rigidity sensing and durotaxis [119–121].

## Cellular state is modulated by the characteristics and stiffness of the contacted material

The adhesive intensity and proliferative ability of cells vary with the stiffness or characteristics of colonized materials [122]. Cells preferentially migrate toward the regions where gradients of adhesiveness increase [123]. SCs maintain a high motility but a low-grade contact to directly migrate along axons in soft mechanical environments but present opposite behaviors in a high-stiffness ECM. Through molecule/integrin receptor pathways and mechanical transduction, SCs can recognize the physical, biological, or chemical properties of ECM. Rac1 switches to regulate the motion of SCs, axonal sorting, and myelination. During axonal sorting and myelination, radial lamellipodia are activated by Rac1 via β1 integrin. Inactivated Rac1 leads to migration of SCs, axon elongation, and myelination arrest. Besides Rac1, Cdc42 is also essential for directional migration and radial axon sorting [124]. By downregulating Rac1, RhoA at a high activity promotes myelination and nuclear elongation, but suppresses migration of SCs at an early stage of axon development [125; 126]). Nuclear elongation is a consequence of the combined action of stress fibers, focal adhesions, and cytoskeletal tension, and is mediated by the RhoA through the Rho/Rock signaling pathway. RhoA and ROCK upregulation increases in matrix elasticity and promotes cell spreading and differentiation [127].

Non-muscle myosin (NMII) is essential for axon ensheathment by SCs. NMII acts on cell morphology and differentiation by altering the elasticity of the environment. NMII responds in an elasticity-dependent manner. Briefly, brain-like matrices (elasticity coefficient of 0.5–1 kPa) are neurogenic, muscle-like matrices (10 kPa) are myogenic, and rigid matrices (20–40 kPa) are osteogenic [128]. However, suppression of NMII fails to perturb cell functions and characteristics [129]. It is proved that the differentiation of SCs is related to actomyosin contractility. cAMP9 stimulates the phosphorylation of MLC and then decreases the contraction of actomyosin to achieve the differentiation of SCs [128]. FAK activation combined with actomyosin contraction further stimulates the spreading and proliferation of SCs on immature basal lamina. However, SCs can independently differentiate without the need to activate FAK after radial sorting and basal lamina maturity [130].

## Durotaxis in peripheral nerve injury

Physical and chemical factors interact directly during peripheral nerve repair, during which the physical property of ECM structurally supports the proliferation and differentiation of nerve cells via intracellular signaling transduction [124] (Xu, Orkwis, & Harris, 2020). SCs become highly sensitive and plastic to adapt to the substrate stiffness for repairing PNI [131]. The interaction between basal lamina deposition and axon maturation modulates the biochemical and mechanical properties of SCs during structural development [12,132]. At the micro-level, basal lamina determines ECM stiffness that varies across different periods of migration and maturation [133].

**Figure 3.**
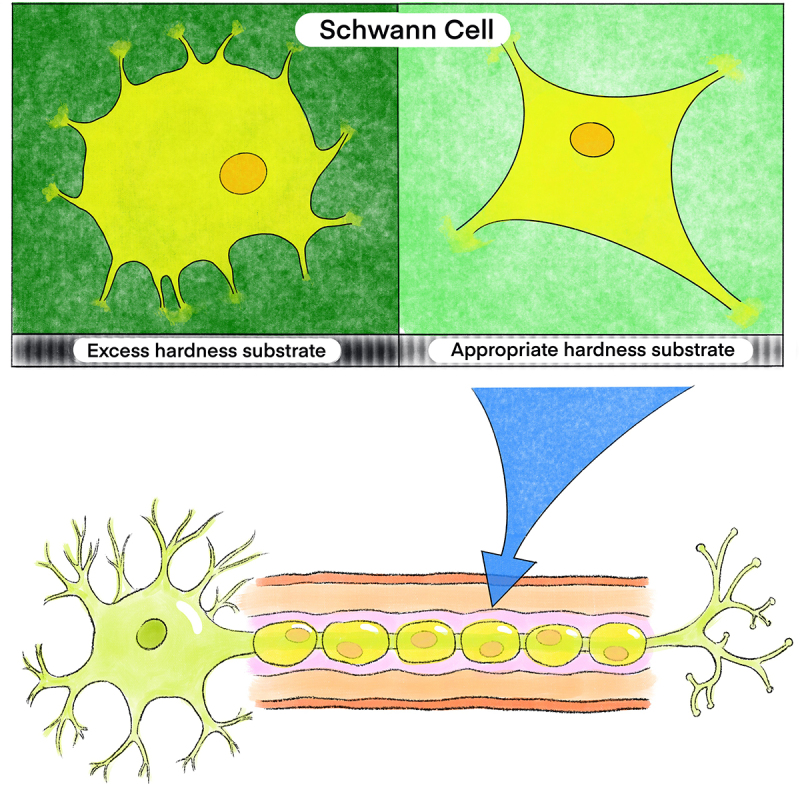
Schwann cells show a multipolar state when they are attached to excess harness substrate materials. However, appropriate hardness induces Schwann cells to transform into a bipolar shape, completing the repair of peripheral nerve injury.

SCs elongate and accelerate the repair of PNI by forming the Bünagner bands [134]. SCs exhibit a multipolar morphology during early development. ECM stiffness guarantees the transformation of multipolar SCs into a bipolar morphology. Previous evidence shows that SCs extend when the stiffness of the surrounding substrate increases [131]. An elastic moduli environment at a high intensity of 4.80 ± 0.29 kPa mold SCs into a multipolar shape; in contrast, the low intensity (1.70 ± 0.09 kPa) forms a bipolar shape [135]. (Figure 3) Within an external environment ranging from 20 kPa to 30 kPa of elastic modules, mature SCs are naturally surrounded by a basal lamina in myelinated adult nerve fibers [128]. Moreover, the stiffness of the adult sciatic nerve consistently increases during development, varying from 5 kPa to 50 kPa. SCs culturing on rigid substrates display a more polygonal morphology [128,131].

Tiered ECM of varying stiffness has shown encouraging influences on the shape, differentiation, and migration of SCs. The direct effect of durotaxis on the migration and function of SCs in repairing PNI need further exploration.

## Conclusion

It remains a challenge to elucidate how SCs sense external cues like chemotaxis, galvanotaxis and magnetotaxis, and transmit information to the cytoskeletal machinery that governs cell translocation. We believe that SCs act depending on the cellular signaling transduction and superficial receptors on their membranes. The mechanisms underlying SC migration and differentiation are parallel but similar [117]. Studies have also emerged to study the effects of non-biological stimulations on the PNI-repairing performance of SCs.

Reparative SCs are well recognized for their potential to promote nerve regeneration. They guide injured axons to their targets, remyelinate regenerated axons, promote neuronal survival, clear myelin debris and prevent neuronal death through various mechanisms. In this article, we described the physical mechanisms that reprogram of SCs into a repair-promoting phenotype, including galvanotaxis, magnetotaxis and durotaxis. In addition, physical stimulations combined with novel biomaterials are more effective in triggering the reparative function of SCs in PNI. We also reviewed the signaling pathways involved in repairing PNI by SCs. In the future, clinical trials are required to validate these effects and mechanisms of SCs.

## Data Availability

Data sharing does not apply to this article as no new data were created or analyzed in this study.
